# Supplementary material for: Patterns of Intron Gain and Loss in Fungi
Source: PLoS Biol. 2004 Nov 30;2(12):e422. doi: 10.1371/journal.pbio.0020422 (PMC532390; doi:10.1371/journal.pbio.0020422)
Supplement: Table S1 — Also available at http://genes.mit.edu/NielsenEtAl/. (4.3 MB ZIP). [file pbio.0020422.st001.zip › NielsenEtAl/html/549.html]

AN0354.1.NCU09817.1.MG05734.1.FG00478.1


```
 CLUSTAL W (1.82) Multiple Sequence Alignments - Introns Inserted


Sequence 1: NCU09817.1	363 aa
Sequence 2: FG00478.1	375 aa
Sequence 3: MG05734.1	370 aa
Sequence 4: AN0354.1	380 aa
Alignment Length: 382 aa
Number Identitical Residues: 246 aa
Alignment Score (without introns) 10792


MG05734.1 	---~----~M0S~FFIPNKNVGNKEESEDWRI1RGYNPLTSPDLLQHEISQTPESRQTVM
NCU09817.1	---~--MS0Q~H~FYIENRNVGNQAESEDWRI1RGYNPLTPPDLLQHEIPQTAESKQTVI
FG00478.1 	---~--MS~S~S0FFIPNKNVGNQADSEDWRI1RGYNPLTPPDLLQHEISQSPESKKTVL
AN0354.1  	MSS0ELII~S~Q~FFLPNPNLGDSNHLEDSRI1RGYNPLTPPNLLQHEIALTEKARQTVL
          	 :: .        *:: * *:*:. . ** ** *******.*:******. : ::::**:

MG05734.1 	QGREEAAAVVNGNDP-MNRLLVIVGPCSIHDPEAALQYCDMLVKEKEKHKDELLIVMRAY
NCU09817.1	NGRKEVVAVVNGTDE-KERLLVVIGPCSIHDPNAALEYCDLLLKEKEKHKDELLIVMRSY
FG00478.1 	EGRNETVAIVNGTDE-KNRLLVVIGPCSIHDPAAALAYCDLLLKEKEKHKDELLIVMRSY
AN0354.1  	QGRAEAIAVAHGTDTDKRRLLVVIGPCSIHDPEMALEYCDRLLKLKEKYKDELLIVMRSY
          	:** *. *:.:*.* . .****::********  ** *** *:* ***:*********:*

MG05734.1 	LEKPRTTVGWKGLINDPDIDNSFKINKGLKMSRQLFVDLTSKGMPIASEMLDTISPQFLA
NCU09817.1	LEKPRTTVGWKGLINDPDIDNSFQINKGLRLSRQLFVDLTSKGMPLASEMLDTISPQFLA
FG00478.1 	LEKPRTTVGWKGLINDPDIDGSFKINKGLRISRQLFVDLTAKGMPIASEMLDTISPQFLA
AN0354.1  	LEKPRTTVGWKGLINDPDIDNSFNINKGLRISRQLFVDLTNKGMPIASEMLDTISPQFLA
          	********************.**:*****::********* ****:**************

MG05734.1 	DLLSAGAVGARTTESQLHRELASGLSFPVGFKNGTDGTLGVAIDAIGAVKHPHHFLSVTK
NCU09817.1	DLLSVGAVGARTTESQLHRELASGLSFPVGFKNGTDGTLGVAIDAIGAVRHPHHFLSVTK
FG00478.1 	DLLSVGAIGARTTESQLHRELASGLSFPVGFKNGTDGSLGVAIDAIGAAQHPHHFLSVTK
AN0354.1  	DCLSVGAVGARTTESQVHRELASGLSFPVGFKNGTDGSLDVAVDAIGSVKHPHHFLSVTK
          	* **.**:********:********************:*.**:****:.:**********

MG05734.1 	PGVVAIVGTVGNEDCFVILRGGKKGTNYDAKSIGEAKEALVKAGLRPRLMVDCSHGNSLK
NCU09817.1	PGVVAIVGTVGNEDCFVILRGGTKGTNYDAKSIAEAKAALEKSGLRQRLMIDCSHGNSNK
FG00478.1 	PGVVAIVGTEGNNDCFVILRGGTKGTNFDAENIALAKEALQKKGVRQRVMIDCSHGNSSK
AN0354.1  	PGVVAIVGTVGNPDCFVILRGGKKGPNYDAASITEAKEKLIAKGLAPRLMVDCSHGNSQK
          	********* ** *********.**.*:** .*  **  *   *:  *:*:******* *

MG05734.1 	DHRNQPKVSHELAEQIKAGEDAIMGVMIESNINEG~SQKVPKEGKAGLKHGVSITDACIS
NCU09817.1	NHKNQPKVAAEIAQQLENGE-------------TG1NQKVPEEGKCGLKYGVSITDACIG
FG00478.1 	DHKNQPKVAADIAGQISKGEDGIMGVMIESNINEG1NQKVPAEGKSGLKYGVSITDACIS
AN0354.1  	DHKNQPKVAAVLAEQIAAGETAIMGVMIESNINEG1NQKVPPEGKAGLKYGVSITDACIN
          	:*:*****:  :* *:  ** .  .   .:. . * .**** ***.***:*********.

MG05734.1 	WQDTVTVLDELAAAVKQRREAAQK0----
NCU09817.1	WEDTVATLDTLAQAVKKRREVLSK~NAAA
FG00478.1 	WEDTISTLNTLAQAVKDRRKLASS~NGA-
AN0354.1  	WEDTESTLETLAKAVATRREKLAP~SS--
          	*:** :.*: ** **  **:     ..
```
